# Assessing the Bioactive Profile of Antifungal-Loaded Calcium Sulfate against Fungal Biofilms

**DOI:** 10.1128/AAC.02551-20

**Published:** 2021-05-18

**Authors:** Mark C. Butcher, Jason L. Brown, Donald Hansom, Rebecca Wilson-van Os, Craig Delury, Phillip A. Laycock, Gordon Ramage

**Affiliations:** a Oral Sciences Research Group, Glasgow Dental School, School of Medicine, Dentistry and Nursing, College of Medical, Veterinary and Life Sciences, University of Glasgow, Glasgow, United Kingdom; b Forth Valley Royal Hospital, Larbert, United Kingdom; c Biocomposites, Ltd., Keele, Staffordshire, United Kingdom

**Keywords:** biofilm, fungal, wound management, antimicrobial agents, *Candida*, joint infections

## Abstract

Calcium sulfate (CS) has been used clinically as a bone- or void-filling biomaterial, and its resorptive properties have provided the prospect for its use as a release mechanism for local antibiotics to control biofilms. Here, we aimed to test CS beads loaded with three antifungal drugs against planktonic and sessile fungal species to assess whether these antifungal beads could be harnessed to provide consistent release of antifungals at biofilm-inhibitory doses. A panel of different fungal species (*n* = 15) were selected for planktonic broth microdilution testing with fluconazole (FLZ), amphotericin B (AMB), and caspofungin (CSP). After establishing planktonic inhibition, antifungal CS beads were introduced to fungal biofilms (*n* = 5) to assess biofilm formation and cell viability through a combination of standard quantitative and qualitative biofilm assays. Inoculation of a hydrogel substrate, packed with antifungal CS beads, was also used to assess diffusion through a semidry material, to mimic active infection *in vivo*. In general, antifungals released from loaded CS beads were all effective at inhibiting the pathogenic fungi over 7 days within standard MIC ranges for these fungi. We observed a significant reduction of pregrown fungal biofilms across key fungal pathogens following treatment, with visually observable changes in cell morphology and biofilm coverage provided by scanning electron microscopy. Assessment of biofilm inhibition also revealed reductions in total and viable cells across all organisms tested. These data show that antifungal-loaded CS beads produce a sustained antimicrobial effect that inhibits and kills clinically relevant fungal species *in vitro* as planktonic and biofilm cells.

## INTRODUCTION

While total joint replacements are among the most successful medical procedures currently undertaken, with good survivorship and low morbidity and mortality (1, 2), the average replacement joint infection rates taken from several arthroplasty registers have gradually increased since 2010 from 0.79% to 0.97% (3). While these numbers may seem low, related prosthetic joint surgery is very common, at an estimated 1 million per year in the United States (4). Infection is a serious postoperative complication and continues to persist despite the use of prophylactic measures (5). Confirmed infection can lead to severe consequences, such as bone destruction, arthroplasty failure, amputation, and even death due to general sepsis (6). This is associated with increased morbidity, hospital length of stay, and overall impact on the health care system, with the total cost in the United States alone estimated at around $1.62 billion (7).

Calcium sulfate (CS) has been used as a biomaterial for clinical applications for many years (8–10). It has stood the test of time due to its well-tolerated nature by the human immune system, relative lack of expense, and resorbable characteristics (11, 12). For these reasons, it has found a dedicated niche in the applications of bone and void filling in dentistry and orthopedics. Perhaps more importantly than this, it has been developed as a tool for the loading of antibiotics and pharmacological agents (10). As the CS steadily dissolves, this provides an avenue for the steady release of loaded antimicrobial agents, which has been shown to be more efficacious than that of other antibiotic-loaded materials, including polymethylmethacrylate (PMMA) (13). The capacity for CS to be used as a release mechanism for local antibiotic loads holds particular relevance within the treatment regimen for diseases such as peri-prosthetic joint infection (PJI), which has been heavily linked with bacterial and fungal colonization in the form of biofilms (14). Indeed, studies have already taken place that investigate the efficacy of antibacterial-loaded CS in revision arthroplasty for postoperative patient management (15–17).

Bacterial colonization of PJI implants is centered around biofilm production (18–20). Biofilms are aggregates of cells encased in an antimicrobial-resistant extrapolymeric barrier. It is the ability of these organisms to form these complex biofilm communities that is strongly correlated with their ability to cause disease in a clinical setting (21). The recalcitrant nature of the biofilm is such that it is harder to tackle with conventional therapy and much more likely to develop over a prolonged instance, such as that of the chronic wound environment (22). Fungal biofilms receive far less notoriety than bacterial biofilms yet are an important clinical entity in biomaterial and wound infection. While PJI is most commonly associated with Gram-positive bacteria, accounting for approximately 50% of cases (23), fungus-driven infection is responsible for 1% of cases and an approximate conservative estimate of $16 million in U.S. annual health care spending (7, 24). It is likely this is due to a deficit in fungal diagnostics and an unrecognized importance of polymicrobial PJI infections (25). This is coupled with the fact that in addition to *Candida* spp., other yeasts and filamentous fungi have been increasingly associated with biofilm infections (25, 26).

Routinely, fluconazole (FLZ) is preferentially used in the management of fungal PJIs and is distributed through oral dosing and subsequent absorption through the gastrointestinal system (27). Due to its safety record, it is favored as a prophylactic therapy and for minor fungal infections. However, increased incidences of tolerance or outright resistance by Candida albicans, as well as innate antimicrobial resistance from other fungal species, limits FLZ’s efficacy (28). Amphotericin B (AMB), a member of the polyene family, is also a systemically distributed antifungal agent. However, its therapeutic success is hampered due to its toxicity (29). Clinically, it is common to address deep-seated or severe tissue infection with a short-term course of AMB and follow up with FLZ once the susceptibility of the invading fungal species has been determined (30). Echinocandins, such as caspofungin (CSP), are the most recent class of antifungals, having been first approved in 2001 as a direct response to increasing resistance to azole drugs (31, 32).

In this study, we assessed the mixing of these antifungal agents with CS. In addition, we assessed whether when these antifungal agents are loaded into CS beads, they have the capacity to inhibit and kill a panel of pathogenic fungi growing as planktonic and biofilm phenotypes. Here, we show for the first time that antifungal agents loaded in CS can successfully control fungal biofilms.

## RESULTS

### Antifungal-loaded beads inhibit planktonic growth.

**FIG 1:**
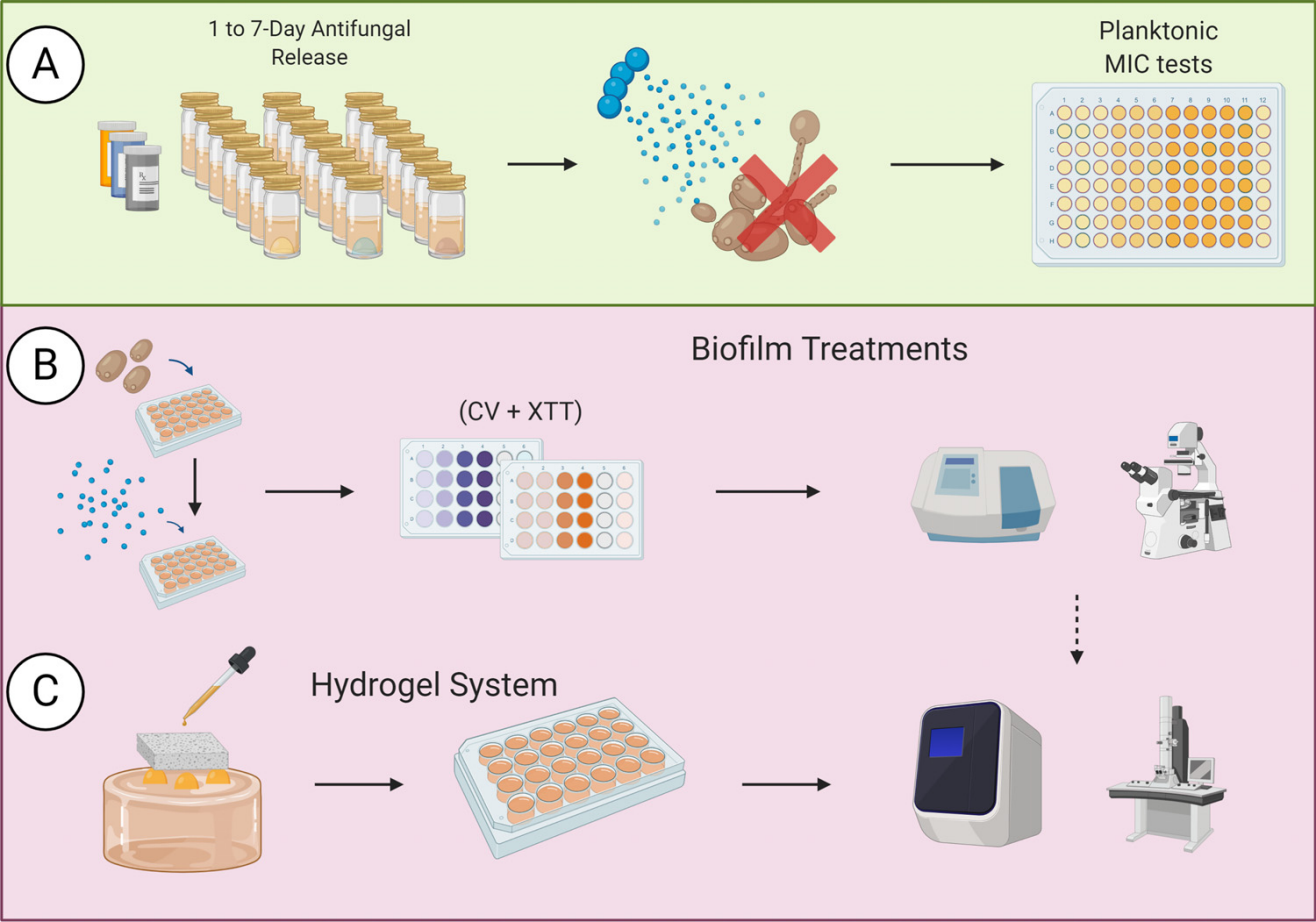
Schematic outline of study methods. (A) Antifungal CS beads loaded with FLZ (80 mg), CSP (35 mg), and AMB (25 mg) are incubated in RPMI for 7 days to test the eluent antifungal effect against planktonic fungal species. (B) Pregrown fungal biofilms are incubated alongside CS loaded with antifungal agents and evaluated via XTT, CV, qPCR, and SEM. (C) The fungal inoculum is introduced to our bespoke hydrogels alongside CS beads to determine the capacity for inhibition of growing biofilm, as evaluated via Live/Dead qPCR and SEM.

All loaded CS beads were shown to effectively release antifungal agents and broadly inhibit the fungi tested (Tables 1, 2, and 3). Yeasts were generally sensitive to FLZ treatment, ranging from 0.3 to 10 μg/ml, with filamentous fungi, though notably Candida auris and Candida tropicalis displayed little to no inhibition (Table 1). For AMB-loaded beads, the results indicate a narrow range of sensitivity for most organisms on all days, ranging between 1.8 and <0.23 μg/ml (Table 2). In CSP-loaded beads, most organisms were found to range between the lowest concentrations tested from 1.2 to <0.15 μg/ml and far below that of the initial concentration of 76 μg/ml. This indicates a lower required concentration to cause inhibition from CSP-loaded beads than observed for AMB-loaded beads when comparing across the panel (Table 3). This is exemplified by C. albicans SC5314, which required concentrations of CSP of less than 0.15 μg/ml, while at times inhibition at concentrations as high as 1.8 μg/ml of AMB was observed. Notably, all 3 drug types released over days 1 to 7 maintained comparable effective concentrations for each of the organisms. A schematic outline of the study can be observed in Fig. 1A.

**TABLE 1:** Broth microdilution of released FLZ over a 7-day incubation period

Species	Strain	FLZ-loaded CS eluent concn (μg/ml)^*a*^						
		Day 1	Day 2	Day 3	Day 4	Day 5	Day 6	Day 7
Candida albicans	SC5314	5	2.5	2.5	2.5	2.5	5	5
ATCC 10231	1.2	0.3	0.6	0.3	0.6	1.2	0.6
Candida glabrata	ATCC 2001	5	5	5	5	5	5	5
Candida auris	NCPF 8978	80	40	40	40	80	80	80
NCPF 8973	20	20	20	80	80	80	80
Malessezia furfur	NCPF 3349
Candida tropicalis	BC064	>160	>160	>160	>160	>160	>160	>160
Candida parapsilosis	NCPF 8334	2.5	2.5	2.5	2.5	2.5	1.2	2.5
Candida haemolunii	DSM 70624	5	10	10	10	2.5	5	5
*Trichosporon* spp.	40	20	40	20	10	10	20
*Rhodotorula* spp.	>160	>160	>160	>160	>160	>160	>160
Aspergillus fumigatus	NCPF 7367	>160	>160	>160	>160	>160	>160	>160
Aspergillus brasiliensis	ATCC 16404	>160	>160	>160	>160	>160	>160	>160
*Rhizopus* spp.
*Scedosporium* spp.

a The table is representative of concentration dilutions extracted from 5 ml of approximately 160 μg/ml eluent of FLZ-loaded Stimulan Rapid Cure beads, diluted 1:1 across a plate with concentrations ranging from 160 to 0.3 μg/ml. Note that there was no inhibition for Malessezia furfur, *Rhizopus* spp., or *Scedosporium* spp.

**TABLE 2:** Broth microdilution of released AMB over a 7-day incubation period

Species	Strain	AMB-loaded CS eluent concn (μg/ml)^*a*^						
		Day 1	Day 2	Day 3	Day 4	Day 5	Day 6	Day 7
Candida albicans	SC5314	0.9	1.8	<0.23	0.45	0.45	0.45	0.9
ATCC 10231	0.9	0.9	<0.23	0.9	0.45	0.45	0.9
Candida glabrata	ATCC 2001	1.8	0.9	0.45	0.45	0.45	0.45	1.8
Candida auris	NCPF 8978	0.9	0.9	0.9	0.9	0.9	0.9	0.9
NCPF 8973	0.9	0.9	0.9	0.9	1.8	0.9	0.9
Malessezia furfur	NCPF 3349	1.8	1.8	0.45	1.8	1.8	1.8	0.9
Candida tropicalis	BC064	3.6	0.45	0.45	0.45	0.45	0.45	<0.23
Candida parapsilosis	NCPF 8334	1.8	0.9	0.9	0.9	0.45	0.45	0.45
Candida haemolunii	DSM 70624	7	3.6	7	7	3.6	3.6	3.6
*Trichosporon* spp.	<0.23	<0.23	<0.23	<0.23	<0.23	<0.23	<0.23
*Rhodotorula* spp.	3.6	0.9	0.9	0.9	0.45	0.45	0.45
Aspergillus fumigatus	NCPF 7367	14	14	14	14	14	7	7
Aspergillus brasiliensis	ATCC 16404	7	7	7	7	7	7	7
*Rhizopus* spp.	1.8	3.6	3.6	3.6	1.8	1.8	1.8
*Scedosporium* spp.

a The table is representative of concentration dilutions extracted from 5 ml of approximately 114 μg/ml eluent of amphotericin B (AMB)-inoculated Stimulan Rapid Cure beads, diluted 1:1 across a plate with concentrations ranging from 114 to 0.23 μg/ml. Note that there was no inhibition for *Scedosporium* spp.

**TABLE 3:** Broth microdilution of released CSP over a 7-day incubation period

Species	Strain	CSP-loaded CS eluent concn (μg/ml)^*a*^						
		Day 1	Day 2	Day 3	Day 4	Day 5	Day 6	Day 7
Candida albicans	SC5314	<0.15	<0.15	<0.15	<0.15	<0.15	<0.15	<0.15
ATCC 10231	<0.15	<0.15	0.3	0.3	0.3	0.3	0.6
Candida glabrata	ATCC 2001	0.6	0.3	0.6	0.6	1.2	1.2	1.2
Candida auris	NCPF 8978	1.2	1.2	1.2	1.2	1.2	1.2	1.2
NCPF 8973	0.6	0.6	0.6	0.6	0.6	0.6	0.6
Malessezia furfur	NCPF 3349	1.2	0.6	0.6	0.6	1.2	1.2	1.2
Candida tropicalis	BC064	<0.15	0.3	0.3	0.3	0.3	0.3	0.3
Candida parapsilosis	NCPF 8334	1.2	1.2	2.3	2.3	4.5	4.5	4.5
Candida haemolunii	DSM 70624	<0.15	<0.15	<0.15	<0.6	<0.3	<0.3	<0.6
*Trichosporon* spp.	>76	>76	>76	>76	>76	>76	>76
*Rhodotorula* spp.	38	38	>76	>76	>76	>76	>76
Aspergillus fumigatus	NCPF 7367	1.2	1.2	1.2	1.2	0.6	19	19
Aspergillus brasiliensis	ATCC 16404	0.3	0.3	0.3	0.3	<0.15	0.6	0.6
*Rhizopus* spp.
*Scedosporium* spp.	19	38	38	38	19	38	19

a The table is representative of concentration dilutions extracted from 5 ml of approximately 76 μg/ml eluent of CSP-inoculated Stimulan Rapid Cure beads, diluted 1:1 across a plate with concentrations ranging from 76 to 0.15 μg/ml. Note that there was no inhibition for *Rhizopus* spp.

### Antifungal-loaded beads are effective against sessile growth.

**FIG 2:**
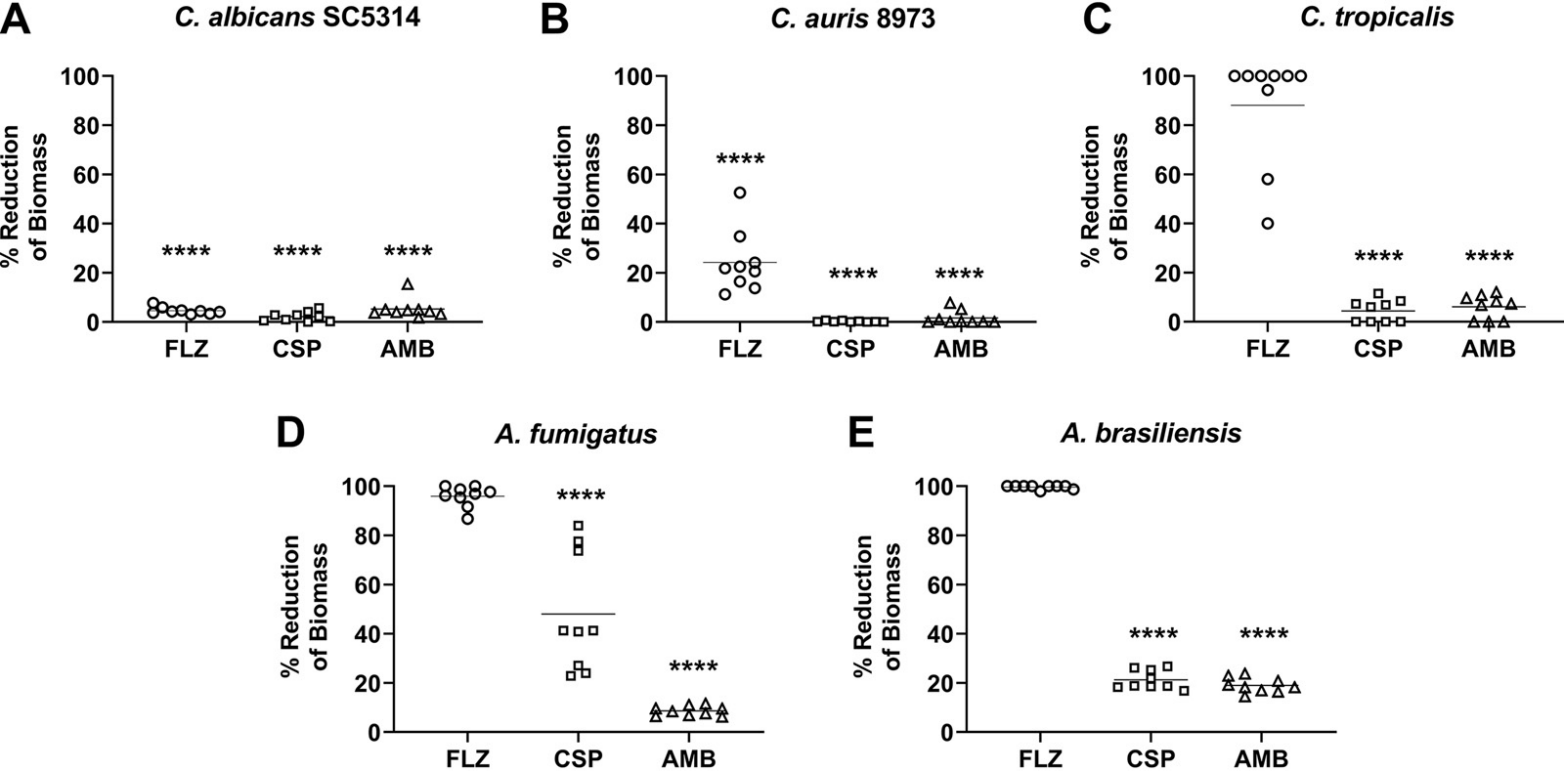
Inhibition of fungal biomass by antifungal CS beads. Shown are results from crystal violet assays of biofilm biomass across 5 fungal species (A to E) 24 h postexposure to FLZ (80 mg)-, CSP (35 mg)-, and AMB (25 mg)-loaded antifungal beads. Results are displayed as a percentage of reduction of biomass compared with unloaded control beads. Significant reductions in biomass (*P* < 0.0001) were recorded across all species (A to E) for CSP- and AMB-loaded beads, while C. tropicalis (C), A. fumigatus (D), and *A. brasiliensis* (E) biofilms displayed resistance to FLZ released from CS beads.

**FIG 3:**
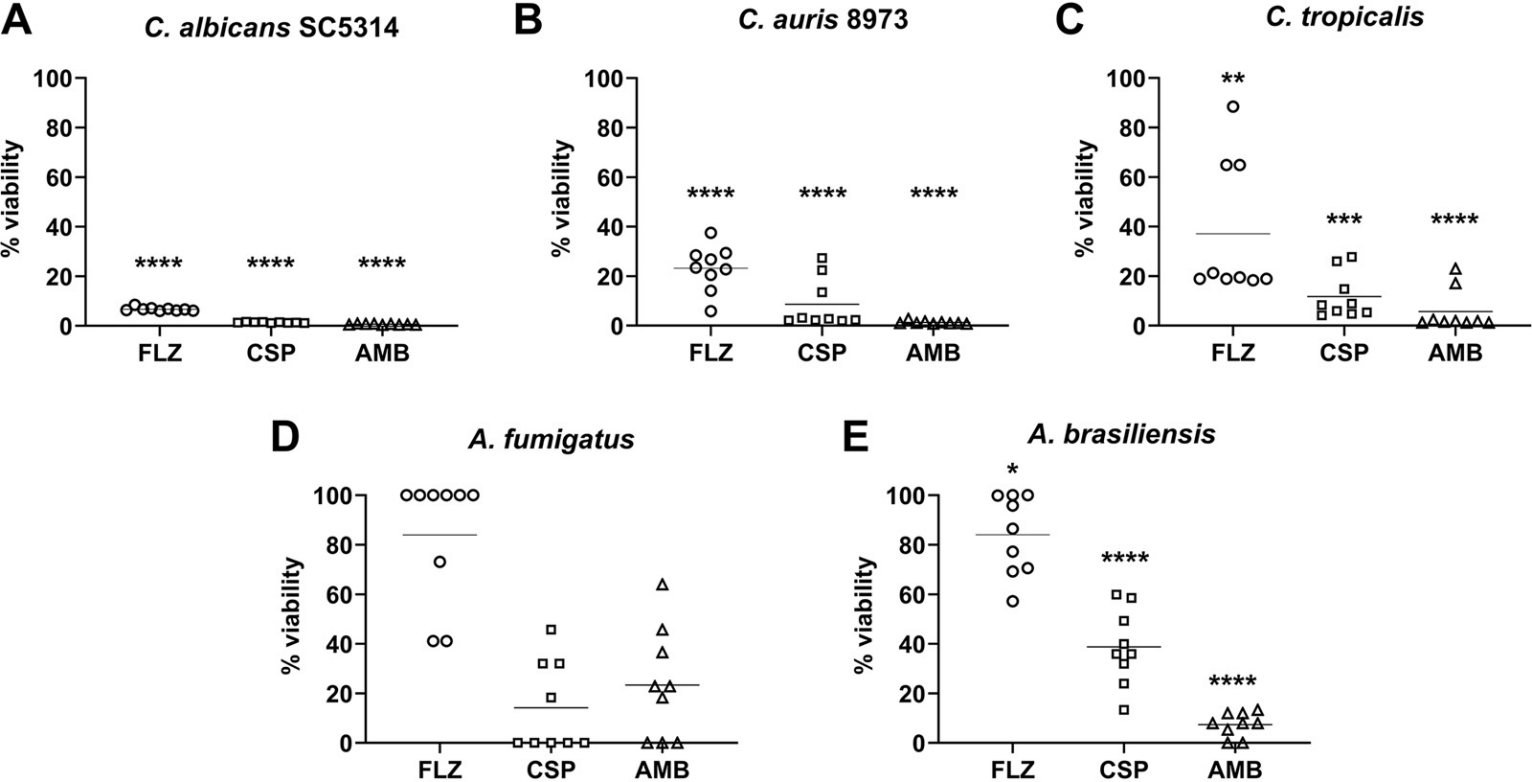
Inhibition of sessile cell viability by antifungal CS beads. Shown are results from a quantitative XTT assay of sessile cell metabolism across 5 fungal species (A to E) 24 h postexposure to FLZ (80 mg)-, CSP (35 mg)-, and AMB (25 mg)-loaded antifungal beads. Results are displayed as percentage of viability compared with biofilms exposed to unloaded control beads. Significant reductions (*P* < 0.0001) in viability were seen for C. albicans (A) and C. auris (B) across all treatments.

Across the 5 organisms selected for biofilm treatment testing, there was a significant reduction in biomass (*P* < 0.0001) after 24 h for CSP- and AMB-loaded beads compared with control beads (Fig. 2). Compared with unloaded control CS, sessile C. albicans growth after exposure to FLZ-loaded beads was reduced by 95%, while C. auris growth was also significantly reduced (*P* < 0.0001), by an average of 76% (Fig. 2). There was no significant reduction in biomass for C. tropicalis, Aspergillus fumigatus, or Aspergillus brasiliensis. Sessile cell viability also corroborates with the biomass data, with reductions in viability across all species, with one exception for CSP- and AMB-loaded beads (Fig. 3). C. albicans sessile viability was reduced by an average of 99% when exposed to CSP- and AMB-loaded beads, while an average reduction of 94% was noted in cells exposed to FLZ-loaded beads (*P* < 0.0001). In C. auris, averages of 92, 99, and 74% reductions in viability were recorded for CSP-, AMB-, and FLZ-loaded beads, respectively. FLZ also recorded drops in viability by an average of 64% for C. tropicalis and 16% for *A. brasiliensis*, despite no reduction in biomass. Despite recorded changes between control and treatments for A. fumigatus, no significance was determined.

**FIG 4:**
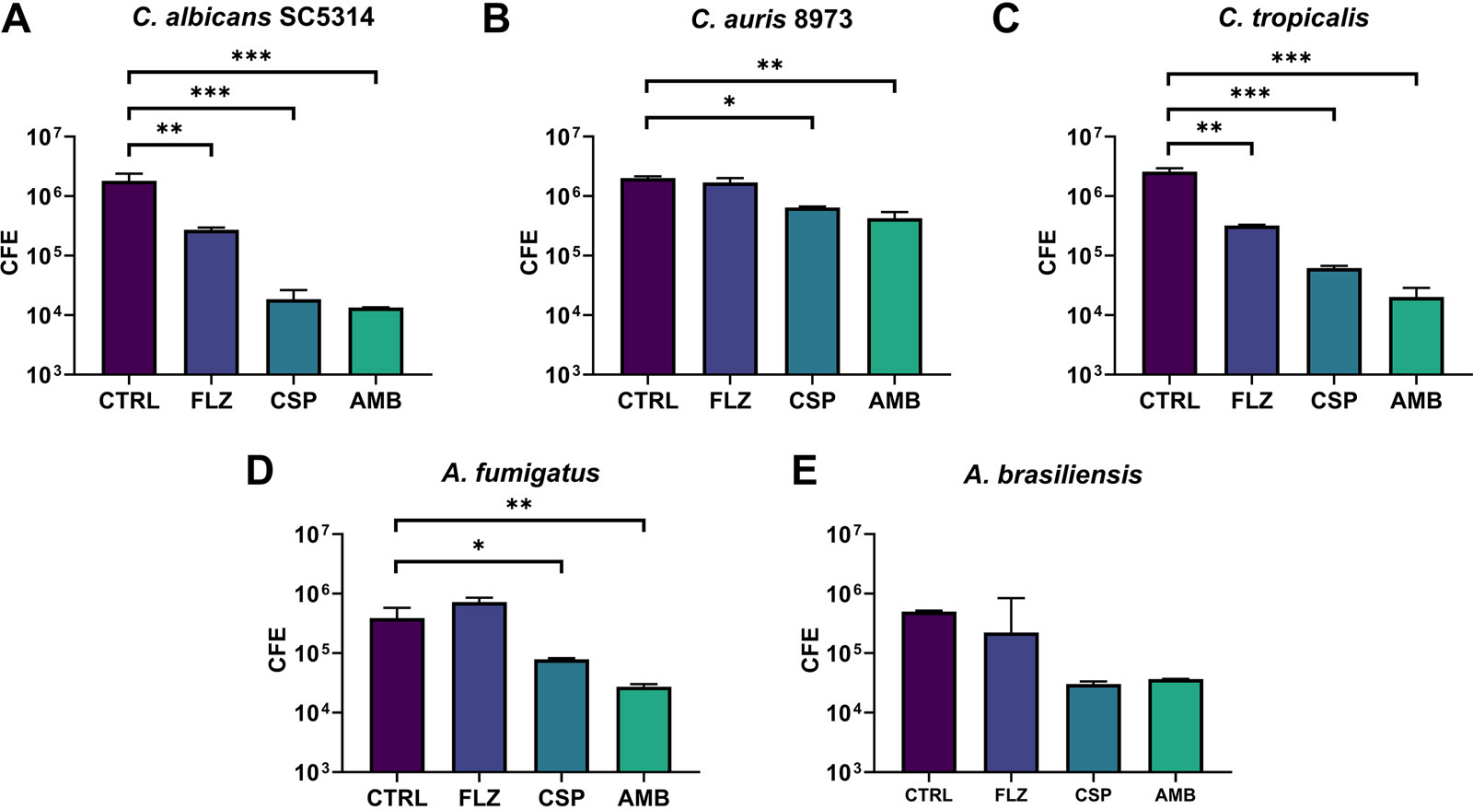
Total qPCR of fungal biofilm treated with antifungal CS after 48 h. Bars represent log CFE of 5 tested fungal isolates (A to E). Results indicate a variable but statistically significant reduction in total CFE for all but one isolate when exposed to CSP- and AMB-loaded beads, while FLZ-loaded beads only proved effective (*P* < 0.005) versus C. albicans SC5314 (A), C. auris 8973 (B), and C. tropicalis (C). In *A. brasiliensis* (E), no significant change (*P* < 0.05) was determined.

**FIG 5:**
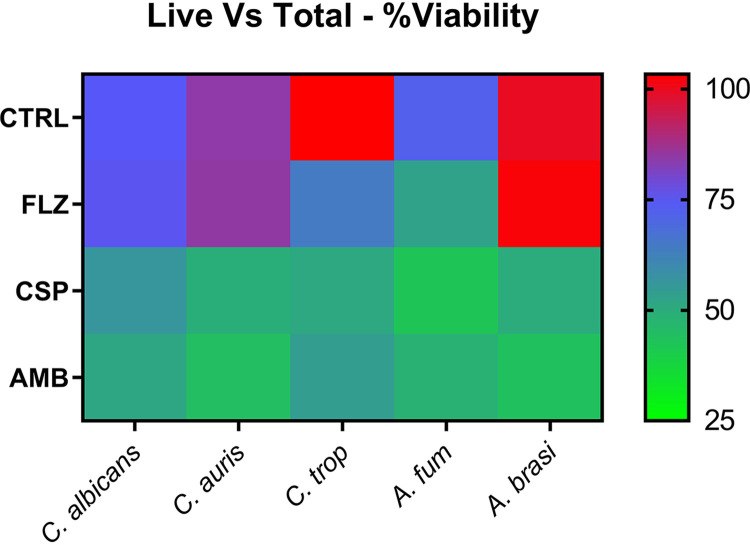
Heat map comparison of viability in comparative live versus total CFE for biofilm treatment. Shown are the results of heat map analysis of live versus total PCR samples from biofilm treatment tests. The scale represents a range from 100% viability (red) to 0% viability (green) when comparing CFEs extrapolated from PMA (live)-treated samples to untreated (total) samples. CTRL, control; *C. trop*, C. tropicalis; *A. fum*, A. fumigatus; *A. brasi*, *A. brasiliensis*.

We also used a molecular-based PCR assessment of fungal biofilm growth. After exposure to antifungal beads, colony-forming equivalent (CFE) values for each fungal isolate were acquired and compared with those of an unloaded control bead to observe drug differences (Fig. 4). The greatest reductions in CFE were observed in C. albicans, with an approximately 140-fold reduction in total cells observed when exposed to AMB-loaded beads (*P* < 0.0005), a 95-fold reduction when exposed to CSP-loaded beads (*P* < 0.0005), and a 7-fold reduction in total cells when exposed to FLZ-loaded beads (*P* < 0.005). While C. tropicalis incubated with CSP-loaded beads produced a 40-fold reduction, AMB-loaded beads produced a 120-fold reduction and FLZ-loaded beads an 8-fold reduction. C. auris observed a 3-fold drop in total CFE on exposure to CSP-loaded beads (*P* < 0.005) and a 4.5-fold reduction when exposed to AMB-loaded beads (*P* < 0.001), with no significant reduction when exposed to FLZ-loaded beads. A. fumigatus also observed a 5-fold decrease in total CFE when exposed to CSP-loaded beads (*P* < 0.005) and a 15-fold reduction in cells when exposed to AMB-loaded beads (*P* < 0.001). *A. brasiliensis* was not significantly reduced when drug-loaded and control beads were compared. Following these analyses, we provided a comparison of the percentage of live cells compared with the total number of cells present in biofilms exposed to CS beads (Fig. 5). These data, displayed as a heat map, indicated that only between 43 and 56% of cells in all samples treated with CSP- and AMB-loaded beads were still viable at the time of DNA extraction. C. albicans, C. auris, and *A. brasiliensis* exposed to FLZ-loaded beads displayed little change in viability compared with control beads, ranging between 75 and 100% viability. It should be noted that, as a result of the increased length of time required for biofilm observations, organism growth kinetics result in a less than 100% viability, even in unaltered control samples. This indicates that while there may be a decrease in total CFE from FLZ-loaded beads (Fig. 4), the majority of cells remaining in a sample exposed to FLZ-loaded beads are still viable, although C. tropicalis and A. fumigatus indicate FLZ-loaded beads as having been a more effective treatment, as only approximately 64 and 53% of the total cells remain viable.

**FIG 6:**
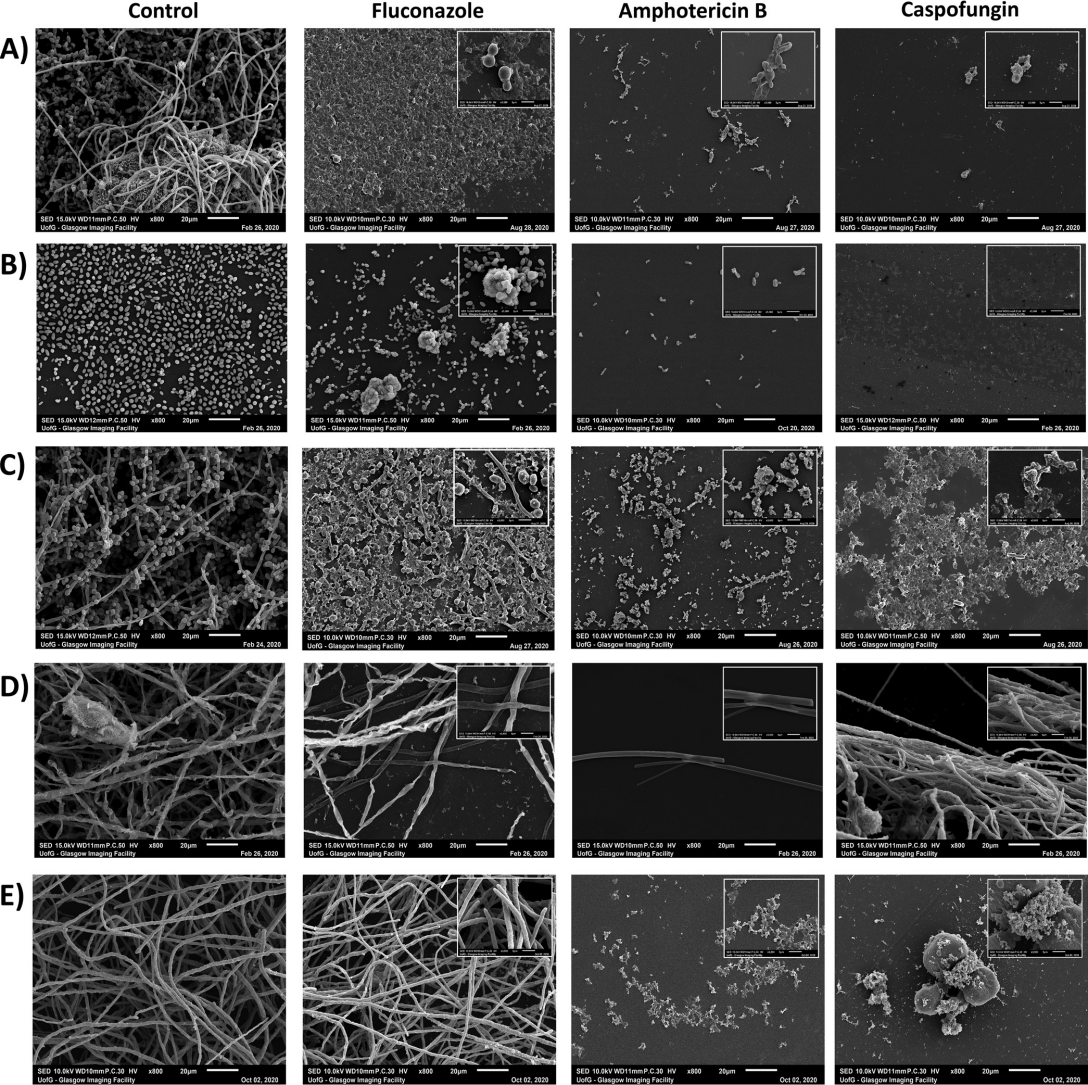
Scanning electron micrography of fungal biofilms post-antifungal-loaded CS exposure. Images depict fungal biofilms challenged with FLZ (80 mg), AMB (25 mg), and CSP (35 mg) beads after 72 h of growth at ×800 magnification and ×3,500 magnification (insets). (A) C. albicans. (B) C. auris. (C) C. tropicalis. (D) A. fumigatus. (E) *A. brasiliensis*.

Scanning electron microscopy (SEM) of biofilms exposed to antifungal beads showed that across all tested organisms, CSP- and AMB-loaded beads produce a pronounced visual effect on the developed biofilm that supports our quantitative analysis (Fig. 6). Visualization supports the quantitative analysis, with C. albicans producing robust biofilm with clear filamentation in the unloaded control sample but obvious destruction of biofilm and malformation of cellular structure against all antifungal-loaded beads (Fig. 6A). C. auris can also be seen to produce a biofilm of morphologically intact cells in the control, which appears only slightly altered when exposed to FLZ-loaded beads, while AMB- and CSP-loaded beads have dramatically reduced the presence of cells on the substrate (Fig. 6B). C. tropicalis produces pseudohyphal structures in the samples exposed to FLZ-loaded beads (inset), reinforcing the atypical resistance profile seen in previous data (Fig. 6C). A. fumigatus and *A. brasiliensis* show expansive filamentation across control samples, with minor observable deformations when exposed to FLZ-loaded beads and severe reduction of biofilm visible in the presence of AMB-loaded beads (Fig. 6D and E, respectively). As with PCR data, A. fumigatus shows only a minor reduction in biofilm visible when exposed to CSP-loaded beads. A schematic outline of this can be observed in Fig. 1B.

### Antifungal-loaded beads are effective at inhibiting fungal biofilms.

**FIG 7:**
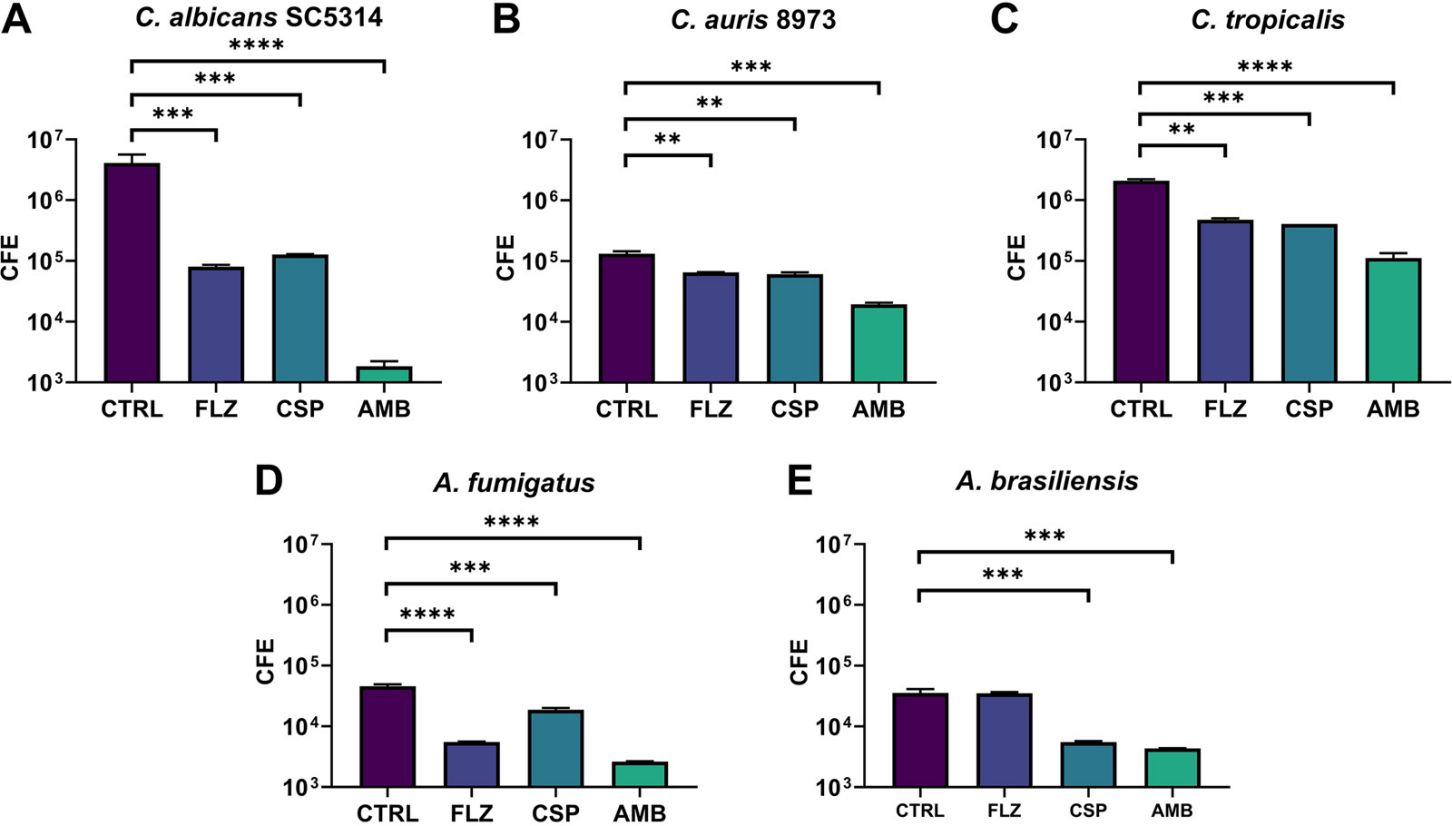
Total qPCR of fungal biofilm inhibited by antifungal CS beads on semidry hydrogels over 48 h. Bars indicate log CFE of 5 tested fungal isolates (A to E), postinoculation on cellulose matrix placed atop hydrogels, including FLZ (80 mg)-, CSP (35 mg)-, and AMB (25 mg)-loaded CS beads. The overall results display a significant reduction of biofilm in samples exposed to AMB-loaded beads. This is also the case for CSP-loaded beads, with the exception of C. auris 8973. FLZ-loaded beads also reduced CFE/ml for all samples, with exception of *A brasiliensis*.

**FIG 8:**
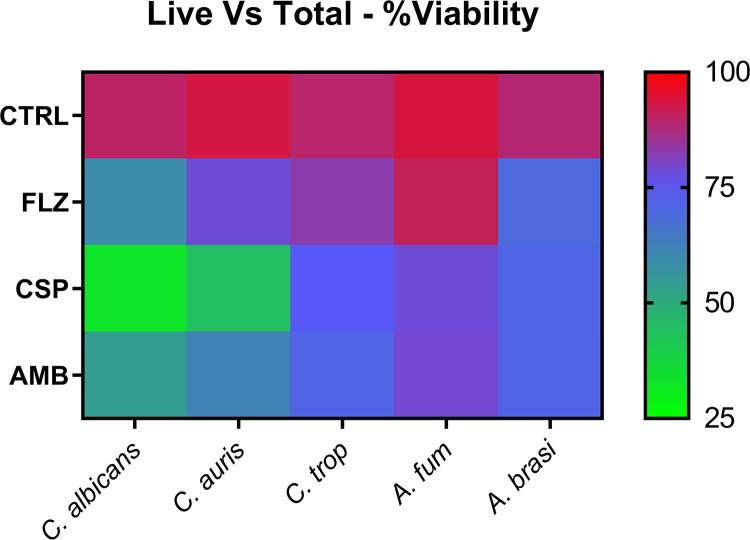
Heat map comparison of viability in comparative live versus total CFE for hydrogel inhibition. Shown are the results from heat map analysis of live versus total PCR samples from hydrogel inhibition tests. The scale represents a range from 100% viability (red) to 0% viability (green) when comparing CFEs extrapolated from PMA (live)-treated samples to untreated (total) samples. CTRL, control; *C. trop*, C. tropicalis; *A. fum*, A. fumigatus; *A. brasi*, *A. brasiliensis*.

Given the positive effects against biofilms in the context of biomaterial-associated infection, we next wanted to assess how loaded CS beads may be used to inhibit biofilm cells in the context of surrounding tissue wound-related infection. Cellulose matrices were inoculated with fungal cells and spores for 2 h to facilitate adherence to the substrata before an overnight incubation with antifungal CS beads within hydrogels (Fig. 7). The data obtained from quantitative PCR (qPCR) analysis of the cellulose matrices showed significant reductions in total CFE. We observed reductions of 2,300-fold in C. albicans (*P* < 0.0001), 18-fold in C. tropicalis, and 17-fold in A. fumigatus, as well as a 7-fold reduction in C. auris and an 8-fold reduction for *A. brasiliensis* (*P* < 0.0005), in samples containing AMB-loaded beads compared with control beads. CSP-loaded beads also indicated significant reductions (*P* < 0.0005) for C. albicans (33-fold), C. tropicalis (5-fold), A. fumigatus (3-fold), and *A. brasiliensis* (6-fold), while C. auris observed a diminished, though still significant, 2-fold reduction (*P* < 0.005). For samples exposed to FLZ-loaded beads, *A. brasiliensis* showed no significant reduction to CFE, A. fumigatus displayed an 8-fold reduction in CFE compared with the control (*P* < 0.0001), while significant reductions were also recorded for all other organisms. When assessing the viability of cells present in each sample (Fig. 8), C. albicans was observed to have the smallest proportions of live cells versus total, with less than 50% live cells in FLZ- and AMB-loaded bead samples and less than 25% in CSP-loaded bead samples. C. auris was also discovered to have less than 50% live cells in CSP-loaded bead samples, while more than 75% of total cells exposed to FLZ-loaded beads remained viable. An outline of the experimental process can be observed in Fig. 1C.

## DISCUSSION

This study aimed to evaluate and provide evidence for the successful, long-lasting effects of antifungal-loaded Stimulan Rapid Cure beads against planktonic and sessile fungal species. As can be observed across the panel of 15 organisms, the concentrations for CSP- and AMB-loaded beads remain consistently effective (required to be diluted approximately 128- to 256-fold) and are found to be largely consistent with values found expressed in other literature (33–37). Where *Candida* species were recorded to be susceptible to doses of AMB-loaded beads ranging between 0.03 and 1 μg/ml and between 0.015 and 0.25 μg/ml for CSP-loaded beads (33). Additionally, previous work has indicated that MIC values for C. auris have a marginally increased effective concentration range for each drug tested in comparison with C. albicans—results found to be consistent with our own (34). Values recorded for FLZ-loaded beads also represented an observed trend in resistance reported in the literature, where multiple sources corroborate the diminished effect of triazoles against multiple species within this panel (38–40). Indeed, through daily replacement of media, the antifungal effect remained consistent with previous publications in which sustained release of antibiotics was observed over a period of 42 days (41). Further investigation without a static system of medium changes, such as under flow, could perhaps provide a more accurate representation of available drug concentration *in vivo*. Nonetheless, we clearly demonstrate a sustained antifungal effect from the CS beads.

With the planktonic data demonstrating proof of success with CS beads releasing antimicrobial doses, the next series of investigations set out to discern if the dose would be sufficient to disrupt an already formed fungal biofilm. The data from this section proved a great success in inhibiting biofilm growth while highlighting already established profiles of resistance among species (38, 40, 42). Biomass and cell viability assays provide a quantifiable assessment of the efficacy of antifungal-loaded CS beads consistent with our initial microdilution data (43). Compared with microdilution data, all organisms, with one exception, displayed sensitivity to CSP- and AMB-loaded beads in both biomass and viability. This is in line with expectations set by initial drug concentrations calculated from the beads. Given that CS beads are in direct contact with the biofilm suspended in liquid media, our estimation is that drug release is many times higher than that which would provide an MIC. The sessile biofilm, which should infer a considerable increase in drug tolerance, is clearly being overcome in the case of AMB- and CSP-loaded beads (44–46). These data make evident that both CSP- and AMB-loaded CS beads have a pronounced effect against a developing fungal biofilm. The success of these investigations is reflected in their similarity to previous studies, where C. albicans biofilm susceptibility to AMB was recorded at a concentration of 8 μg/ml (42), while CSP susceptibility in biofilm has been reported to be between 0.5 and 4 μg/ml (47). When evaluating FLZ-loaded beads, previously reported evidence of biofilm-related drug tolerance becomes clear (48). While concentrations of released FLZ from CS beads were sufficient to inhibit planktonic cells, this was not the case for tested biofilms. Indeed, this was also reflected in previous investigations where it was reported that a panel of 30 *Candida* isolates in biofilm growth were resistant to concentrations of FLZ above 256 μg/ml, twice the concentration available in our loaded FLZ beads (42).

The morphology of fungal cells and hyphae shown in control bead-exposed samples can be seen to consistently match those in electron micrographs that have been published previously (47, 49–51). Those biofilms exposed to AMB- and CSP-loaded beads also follow the pattern in previously published occurrences, having disrupted and deflated cell wall structure where cells are still present upon the substrate (47, 52). These images provide insight and adequate reinforcement of the conditions displayed in our biomass and cell viability assays. However, the nature of these images is such that it provides only a very small representation of the sample as a whole, so it is insufficient for forming conclusions of the efficacy of treatment by itself.

We set out to determine a quantifiable colony-forming equivalent (CFE) for total cells present in a sample and specifically for live cells present in a sample. This provided a much clearer representation of how effective drug release from CS beads was at diminishing biofilms of each organism. Indeed, significant fold reductions were seen for almost every organism. Diminished sensitivity to treatment in C. auris could be related to phenotypic switching of a nonaggregate to aggregative cell morphology, which has been previously shown to infer a higher profile of antimicrobial resistance (50, 53). Additionally, *A. brasiliensis* also shows no significance in CFE reduction compared with an unloaded control bead, although there is a reduction of CFE in each treatment type. The susceptibility profile of *Aspergillus* spp. has been seen previously to lack susceptibility to drugs such as azoles and echinocandins (46, 54).

The biofilm treatment studies discussed above provided evidence of the success of Stimulan Rapid Cure-loaded CS beads at disrupting established fungal biofilms during simultaneous incubation. This may be improved upon through recognition of other systems for imitating *in vivo* growth conditions that may have developed over time, such as those of the chronic wound under conditions like diabetic foot ulceration (DFU). While this also addresses the concern of prophylactic prevention of biofilm formation under conditions such as PJI, where CS beads have been investigated clinically (55), and can be integrated into such a system. Previous studies have elucidated the need for an *in vitro* methodology for biofilm testing that more reliably mimics *in vivo* conditions (56). While the hydrogel system has undoubtedly been a key representative in investigations relating to the marriage of antimicrobials, hydrogels, and wound care (57–59), it has perhaps been underutilized in relation to fungal biofilms. The results from our hydrogel model analysis determined a similar trend in CFE reduction in the developing biofilm to that presented in the biofilm treatment model. This was able to provide valuable insight into the impact of antifungal-loaded CS on the fungal biofilm in a more complex system than that of a traditional plate model. One notable drawback to these investigations of the developing biofilm was the inability to differentiate spore destruction as a result of antifungal exposure, which is of clinical relevance in the case of possible infection recurrence and reestablishment of biofilm. With this in mind, while this study has helped bring to light a number of key interactions between antifungal-loaded CS and pathogenically relevant fungi, there remains a larger picture of interspecies and interkingdom interactions to be considered. Recently, our group has recorded the merit of considering fungi in relation to complex wound biofilms (60, 61), where the inclusion of fungal communities in these models may be key to replicating and investigating key clinical concerns in the treatment of chronic wounds (62). Having observed the effect of CS beads on bacterial biofilms, through previously published work and with our own observations described herein, the next logical step is to investigate their impact on polymicrobial, interkingdom biofilms (13).

In conclusion, antifungal-loaded CS beads provide a mechanism for release of sustained, biofilm-inhibitory doses of antifungal compounds. We have demonstrated the effective disruption of clinically relevant and emerging fungal pathogens such as C. albicans and C. auris over a series of time points. Overall, this implies great potential for further clinical development of antifungal-loaded CS for use in the prevention and possible treatment of fungus-driven wound infections.

## MATERIALS AND METHODS

### Microbial growth.

A panel of 15 fungal species comprising Candida albicans ATCC MYA-2876 and 10231, Candida glabrata ATCC 2001, Candida auris NCPF 8978 and 8973, Candida tropicalis BC064, Candida parapsilosis NCPF 8334, Candida haemolunii DSM 70624, Malassezia furfur NCPF 3349, *Rhodotorula* spp., *Trichosporon* spp., Aspergillus brasiliensis ATCC 16404, Aspergillus fumigatus NCPF 7367, *Scedosporium* spp., and *Rhizopus* spp. was assembled for this study. Strains were maintained on Sabouraud dextrose (SAB) agar (Oxoid, Hampshire, United Kingdom) and refrigerated at 4°C prior to proliferation in yeast-peptone-dextrose (YPD; Sigma-Aldrich, Dorset, United Kingdom) in a 200-rpm shaking incubator overnight at 30°C for *Candida*, *Malassezia*, *Trichosporon*, and *Rhodotorula* species. Spores of the pore-forming fungi *Aspergillus*, *Rhizopus*, and *Scedosporium* were harvested using a previously described method for storage at 4°C (63). Spores and yeast cells were pelleted through centrifugation at 3,000 × *g* and washed twice with phosphate-buffered saline (PBS). Cells and spores were then counted using a Neubauer hemocytometer and standardized to a working concentration for further experimentation.

### Preparation of Stimulan Rapid Cure calcium sulfate beads.

A prepackaged kit containing 9.6 g of pharmaceutical-grade CS α-hemihydrate powder (CS-CSH) (Stimulan Rapid Cure; Biocomposites, Ltd., United Kingdom) was mixed with 3.1 ml of deionized water to create unloaded CS beads. To prepare antifungal-loaded beads, an additional 35 mg of CSP acetate powder or 80 mg of FLZ powder was added to the CS-CSH mixture prior to mixing. For AMB, 25 mg was added to a CS mixture with 3.5 ml of 0.9% saline solution. Drug concentrations were selected primarily based on daily dosage of intravenous drugs (CSP and FLZ) or the maximal concentration of drug that could be added to CS-CSH while still allowing it to fully set (AMB). These combinations were then mixed for approximately 30 s until a smooth paste had formed and then spread into a 6- or 3-mm pellet mold and allowed to harden for a minimum of 30 min, until set, under aseptic conditions. The beads were then weighed in order to determine their antifungal load. The 6-mm AMB, CSP, and FLZ beads were averaged at 0.143, 0.138, and 0.127 g, respectively. The antifungal loads per bead were determined to be 0.56 mg of AMB, 0.38 mg of CSP, and 0.79 mg of FLZ.

### Planktonic fungal susceptibility testing.

To first test whether each antifungal agent was successfully released from the CS beads consistently over time, a planktonic broth microdilution test was performed as a surrogate marker of antifungal release. A 1- by 6-mm antifungal-loaded CS bead was incubated in 5 ml of Roswell Park Memorial Institute 1640 (RPMI 1640) over a course of 7 days, with supernatant removed daily to assess the effective concentration required to inhibit planktonic growth as a proxy measure for antifungal release. There were 7 different supernatants, representing each of the 7 days, for each antifungal agent. For time points not being immediately assessed, supernatant was changed daily to evaluate any drift in concentration across the 7 days. Fungal cells were then standardized to 2 × 10^4^ CFU/ml, and fungal spores were standardized to 2 × 10^3^ CFU/ml, as previously described, and broth microdilution testing was performed on each antifungal supernatant according to Clinical and Laboratory Standards Institute (CLSI) guidelines for yeasts and spores (64, 65). Effective concentrations were assessed visually for reduction in turbidity for each antifungal.

### Sessile fungal susceptibility testing.

For biofilm treatment studies, all yeast cell organisms were standardized to 1 × 10^6^ CFU/ml, and spore-forming organisms were standardized to 1 × 10^5^ CFU/ml according to standardized methods (43, 66, 67). These were then incubated in 24-well, flat-bottom plates containing 500 μl of RPMI for 24 h, washed in PBS, and then 1- by 6-mm CS antifungal-containing beads and unloaded control beads were added to respective wells to assess biofilm killing. These were incubated for 1, 3, or 5 days, with daily medium changes, at 37°C. All CS beads were removed using forceps under aseptic conditions, and the resultant biofilms were washed twice with PBS. Immediately after washing, XTT [2,3-bis(2-methoxy-4-nitro-5-sulfophenyl)-2H-tetrazolium-5-carboxanilide salt; Sigma-Aldrich, United Kingdom] metabolic reduction assay was used to assess cell viability, as described previously (43). In addition to this, the antifungal effect on fungal biofilm biomass was determined through a crystal violet (CV) assay (68). Biofilms were allowed to dry at ambient temperature for 24 h before being stained with 0.1% (wt/vol) CV solution for 20 min then washed gently 3 times to remove excess stain. Plates were then inverted and dried at room temperature before bound CV was solubilized using 90% ethanol. The contents of each well were mixed and then transferred, in triplicate, from each 24-well plate to a corresponding 96-well, round-bottom plate. Both XTT and CV assays were assessed using a FLUOstar Omega plate reader at 492 and 570 nm, respectively (BMG Labtech, Aylesbury, United Kingdom). All assays were performed in triplicate on three separate occasions.

### Scanning electron microscopy.

Selected fungal biofilms were grown in RPMI on 13-mm Thermanox coverslips (Fisher Scientific, Loughborough, United Kingdom) at 1-, 3-, and 5-day time points alongside 6-mm antifungal-loaded CS beads of FLZ, AMB, and CSP and an unloaded control bead. Following the incubation period, biofilms were washed 3 times with PBS and then fixed in a solution of 2% glutaraldehyde, 2% paraformaldehyde, 0.15% alcian blue powder, and 0.15 M sodium cacodylate. Next, this was counterstained with uranyl acetate for 1 h, and a gradient dehydration from 30% to 100% ethanol was performed. Finally, a critical point drying step was performed in hexamethyldisilazane (HMDS) in accordance with a previously described protocol (69). Samples were then sputter-coated with gold/palladium and visualized using a JEOL JSM-6400 scanning electron microscope (JEOL, Ltd., Hertfordshire, United Kingdom).

### PCR quantification after biofilm treatment.

Biofilms were grown on 13-mm Thermanox coverslips and treated as described above. Following treatment, they were transferred to 5-ml Bijou bottles containing 1 ml PBS and sonicated at 35 kHz for 10 min to release the biofilm from the coverslip. These 1-ml samples were split into two 500-μl aliquots in nuclease-free Eppendorf tubes (ThermoFisher, Ltd., Renfrew, United Kingdom), with half of the samples set aside for total cell qPCR and the other half treated with propidium monoazide (PMA; ThermoFisher, Ltd., Renfrew, United Kingdom), a DNA intercalating dye that renders DNA not within a membranous structure as insoluble and prevents its detection during PCR (70–72). In brief, 50 μM PMA was added to each sample before the sample was wrapped in aluminum foil and incubated in the dark for 10 min. These were then transferred to a bed of ice before being exposed to a 650-W halogen lamp for 2.5 min on each side to promote PMA intercalation. Both PMA and untreated samples were then transferred to bead-beating tubes containing 0.5-mm acid-washed glass beads that were used to manually disrupt the fungal cell wall. This was to facilitate DNA extraction using 3× 30-s cycles (Fisherbrand Bead Mill homogenizer [ThermoFisher, Ltd., Renfrew, United Kingdom]). Next, DNA purification was carried out using a MasterPure yeast DNA purification kit according to the manufacturer’s instructions (Lucigen, Middleton, WI, USA). The DNA concentration was quantified with a NanoDrop 1000 spectrophotometer (Life Technologies, Paisley, United Kingdom). The colony-forming equivalent (CFE) count was determined using 18S gene primers specific to fungal 18S rRNA (forward primer 5′-CTCGTAGTTGAACCTTGGGC-3′ and reverse primer 5′-GGCCTGCTTTGAACACTCTA-3′), and CFE were extrapolated from threshold cycles (*C_T_*) using the 2^−Δ^*^CT^* method and quantified using the 2^−ΔΔ^*^CT^* method, outlined as described previously (73). A standard curve comparison method for fungal CFU ranging from 1 × 10^3^ to 1 × 10^8^ CFU/ml by quantitative PCR (qPCR) was then undertaken (74). The following thermal profile was used: a holding stage of 50°C for 2 min, followed by a denaturation stage of 95°C for 10 min, and then 40 cycles of 95°C for 3 s and 60°C for 15 s, using the StepOne real-time PCR system and software (Life Technologies, Paisley, United Kingdom).

### Biofilm inhibition.

To assess whether loaded CS beads could inhibit biofilm growth within more translatable environmental parameters than those of a traditional plate model, an alternative hydrogel model was used that was augmented from our previously published wound model (60). This consists of 10% 3-sulfopropyl acrylate potassium salt, 0.95% 22% (vol/vol) polyethylene glycol deacrylate (PEG), 0.01% (vol/vol) 1-hydroxycyclohexyl phenyl ketone, and 50% heat-inactivated horse serum (HS) (ThermoFisher, Loughborough, United Kingdom) mixed with sterile water to make up the final volume. One milliliter of hydrogel solution was added to each well of a 24-well, flat-bottom plate before being polymerized under a 366-nm UV lamp (Camag, Hungerford, United Kingdom) for 30 min. One-square-centimeter squares of cellulose matrix (IPS Converters, Oldham, United Kingdom) were added to universals containing 10 ml of selected fungi, standardized to 1 × 10^6^ cells/ml, and incubated for 2 h at 30°C in a shaking incubator set to 200 rpm to allow fungal cell and spore adherence. These inoculated matrices were then placed upon a “bed” of 4- by 3-mm antifungal-loaded CS beads and unloaded control beads and incubated for 24 h. After incubation, the cellulose matrices were transferred to 5-ml Bijou bottles containing 1 ml of PBS, before being sonicated to remove adhered cells. A PCR-based quantification of adherent fungi was carried out as described above.

### Statistical analysis.

Statistical analyses and graph production were performed using GraphPad Prism (version 8.4.3; GraphPad Software, Inc., La Jolla, CA). Ordinary one-way analysis of variance (ANOVA) was applied to compare means of multiple drug effects among fungal samples. CFU and CFE/ml were log transformed before statistical analyses took place. Statistical significance was achieved at *P* < 0.01.
